# Detection of polymyxins resistance among *Enterobacterales*: evaluation of available methods and proposal of a new rapid and feasible methodology

**DOI:** 10.1186/s12941-023-00618-7

**Published:** 2023-08-10

**Authors:** Gabriela da Silva Collar, Natália Kehl Moreira, Priscila Lamb Wink, Afonso Luís Barth, Otávio Hallal Ferreira Raro, Cícero Dias, Adriano de Lima Machado, Mariana Preussler Mott, Juliana Caierão

**Affiliations:** 1 Laboratório de Pesquisa em Bacteriologia Clínica (LaBaC), Faculdade de Farmácia, Universidade Federal do Rio Grande do Sul, Porto Alegre, Brasil; 2 Programa de Pós-Graduação em Ciências Farmacêuticas, Faculdade de Farmácia, Universidade Federal do Rio Grande do Sul, Porto Alegre, Brasil; 3 Laboratório de Pesquisa em Resistência Bacteriana (LABRESIS), Hospital de Clínicas de Porto Alegre, Porto Alegre, Brasil; 4 Departamento de Ciências Básicas da Saúde, Universidade Federal de Ciências da Saúde de Porto Alegre, Porto Alegre, Brasil; 5 Laboratório Central de Análises Clínicas, Grupo Hospitalar Conceição, Porto Alegre, Brasil; 6 Laboratório de Análises Clínicas, Hospital Ernesto Dornelles, Porto Alegre, Brasil

**Keywords:** Polymyxins, Colorimetric test, *Enterobacterales*, Polymyxin broth disk elution

## Abstract

**Background:**

Fast and accurate detection of polymyxins resistance is necessary as they remain the last resources to treat infections caused by Carbapenem-resistant *Enterobacterales* in many regions. We evaluated the rapid colorimetric polymyxin B elution (RCPE) and developed its miniaturized version, RCPE microelution (RCPEm), aiming to detect polymyxins resistance among *Enterobacterales*.

**Methods:**

The methodologies consist of exposing the bacterial population in a solution (NP solution) where polymyxin B disks were previously eluted to obtain a concentration of 2 µg/mL for RCPE and 3 µg/mL for RCPEm.

**Results:**

Two hundred sixty-seven *Enterobacterales* were evaluated, 90 (33.7%) resistant to polymyxin B by broth microdilution. It was observed 0.6% of major error (ME) by RCPE, with a specificity of 99.4%. The miniaturized version (RCPEm) presented the same ME and specificity values, but slightly higher sensitivity (97.8% vs. 95.6%) with 2.2% of very major error (VME).

**Conclusions:**

RCPE and RCPEm proved to be useful alternatives to determine polymyxin B susceptibility in clinical microbiology laboratories, presenting low cost, being easy to perform, and demanding short incubation time.

**Supplementary Information:**

The online version contains supplementary material available at 10.1186/s12941-023-00618-7.

## Background

Antimicrobial resistance is a subject of major concern to public health worldwide. Carbapenem-resistant *Enterobacterales* (CRE) is recognized, by the World Health Organization (WHO), as “priority 1: critical” for the research and development of new antimicrobials [1]. Indeed, therapeutic options available to treat infections caused by CRE are limited; as a consequence, they are associated with prolonged hospitalization, and high mortality rates [2–4].

In these cases, polymyxins (colistin and polymyxin B) and the new combinations of β-lactams/β-lactamase inhibitors are important choices [3]. However, due to the inefficiency of β-lactamase inhibitors against some carbapenemases, and due to the unavailability of these new combinations in some countries (as well as their high cost), polymyxins-centered therapeutic regimens remain an important and/or the last resort in many regions [5–8].

Although polymyxins resistance rates remain low in some countries, there is a steady increase in others, coinciding with the increase in the frequency of CRE [9–13]. In this context, rapid and accurate detection of polymyxins resistance is crucial [14]. However, disk diffusion and concentration gradient strips are not accurate enough for this purpose [15–18]. Indeed, the Clinical and Laboratory Standards Institute (CLSI) and the European Committee on Antimicrobial Susceptibility Testing (EUCAST) established broth microdilution (BMD) as the reference methodology to determine polymyxins resistance [19, 20]. Despite its reliability, BMD presents important disadvantages, mainly, the requirement of a prolonged incubation (16-20 h) [18].

Thus, new methodologies better adapted to current needs have been developed. In 2016, Nordmann, Jayol & Poirel [21] described the Rapid Polymyxin NP (RPNP), which detects polymyxins resistance in up to 4 h, based on glucose metabolism by microorganisms in the presence of a defined concentration of antibiotic and a pH indicator (NP solution). Although broadly evaluated with excellent performance overall [22–31], RPNP is not endorsed by CLSI or EUCAST. In 2019, Simner and colleagues [32] developed the colistin broth disk elution (CBDE), which became recommended by CLSI [20] for the detection of colistin resistance. So far, few publications evaluated this technique, but studies demonstrate good results [32–35]. It should be noted that, to our knowledge, only two studies have been performed using polymyxin B (PBDE) instead of colistin so far [36, 37]. The main advantage of CBDE/PBDE is the use of antibiotic disks instead of powder, reducing costs, which may be an issue in low-incoming countries. On the other hand, the required incubation is as long as BMD, which is a notable disadvantage.

In 2021, Ngudsuntia et al. [38] proposed a modification in RPNP, presenting a methodology that determines colistin resistance after elution of a colistin disk in the NP solution, providing results in up to 4 h. It had satisfactory results but the number of isolates evaluated by the aforementioned authors was limited, encouraging further studies to better determine its performance. Indeed, an alternative methodology that could combine the advantages of RPNP and CBDE/PBDE would be valuable for clinical microbiology laboratories.

Here, we evaluated the rapid colorimetric polymyxin B elution (RCPE), which is based on Ngudsuntia and coworkers [38]. Also, we established the miniaturized version, rapid colorimetric polymyxin B microelution (RCPEm), to detect resistance quickly and in a low-cost manner. We compared the performance of these tests, as well as those previously published, RPNP and PBDE, to the reference method, BMD.

## Methods

### Bacterial strains and determination of minimum inhibitory concentration (MIC)

Two hundred sixty-seven carbapenem-resistant *Enterobacterales* recovered from clinical specimens of patients attended in four different hospitals of Porto Alegre city, Southern Brazil, from 2015 to 2022 (supplementary table) were included. In two of these hospitals, isolates were recovered consecutively, and, in the other two, isolates were selected by convenience, being resistance to carbapenem the inclusion criteria. The study was approved by the local research ethics committee. *E. coli* ATCC 25922 and *Morganella morganii* (intrinsically resistant to polymyxins, MIC > 64 µg/mL) were used as negative (susceptible) and positive (resistant) controls, respectively. To determine polymyxin B MIC, BMD was performed and results were interpreted according to CLSI [20] guideline: MIC ≥ 4 µg/mL indicated resistance.

### Polymyxin B disk elution (PBDE) and rapid polymyxin NP test (RPNP)

All isolates were submitted to PBDE and RPNP. PBDE was performed as described by Cielo et al. (2020) [36]. The presence of turbidity in tubes without and with antibiotics (2 µg/mL) indicated a positive result (resistant isolate). RPNP was done according to Nordmann, Jayol & Poirel (2016) [21], testing polymyxin B instead of colistin, in a concentration of 3.75 µg/mL. Plates were read visually after each 1 h of incubation, for up to 4 h. Color change from orange to yellow indicated resistance to polymyxin B, which could have been observed at any time (1, 2, 3 or 4 h). If no color change was observed after 4 h, the isolate was considered negative.

### Rapid colorimetric polymyxin B elution (RCPE and RCPEm)

**Fig. 1:**
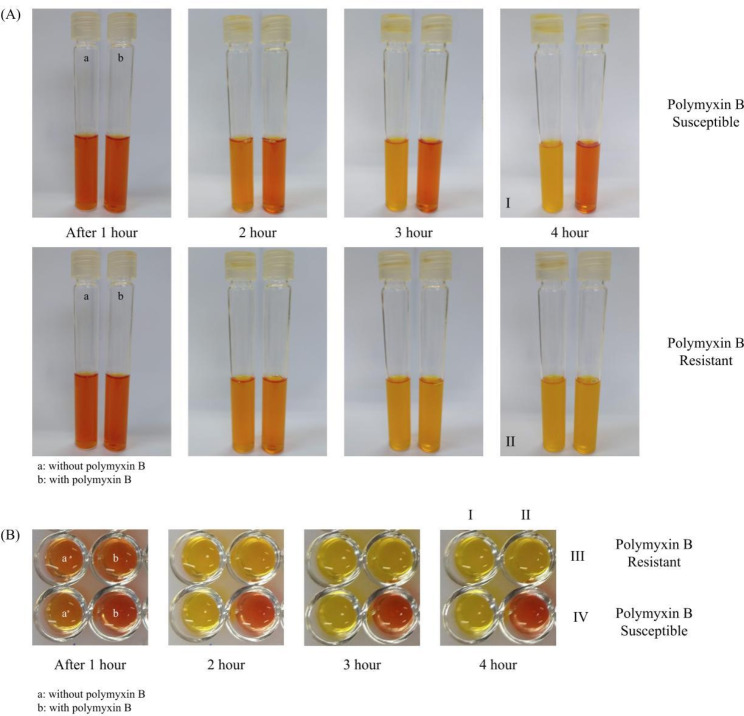
Representative results of the RCPE and RCPEm tests (**A**) Representative results of Rapid colorimetric polymyxin B elution (RPCE) test at every hour of reading, for up to 4 h, with growth being evidenced from color change (orange to yellow). For each image of two tubes, the tube on the right contains the eluted polymyxin B disk, reaching a concentration of 2 µg/mL. I: Susceptible isolate due to permanence of orange color in a tube containing antibiotic disk. II: Resistant isolate due to color change of tube containing antibiotic disk. (**B**) Rapid colorimetric polymyxin B microelution (RCPEm) test results at each hour of reading for up to 4 h. The color change of the wells from orange to yellow indicates bacterial growth. I: Antibiotic-free column of wells (growth control). II: Column of wells with NP solution where antibiotic disks were previously eluted, resulting in a final concentration of 3 µg/mL of polymyxin B. III: Resistant isolate due to color change in the well containing the antibiotic. IV: Sensitive isolate due to continuity of orange staining of an antibiotic-containing well

The test was performed based on Ngudsuntia et al. (2021) [38], with some modifications, in order to (i) adapt volumes to the final concentrations of polymyxins desired, as colistin and polymyxin B disks have different quantity of antibiotic, and (ii) to better standardize inoculum, ensuring reproducibility. For each isolate, we used two tubes, containing 14 mL of NP solution (10% anhydrous glucose, cation-adjusted Mueller-Hinton broth [Sigma-Aldrich, USA] and phenol red [Sigma-Aldrich, USA]) [21] in each. In one tube, a 300 IU polymyxin B disk (Oxoid, United Kingdom) was added to elute in order to reach a final concentration of 2 µg/mL. The tube was kept at room temperature for 30 min for elution. Then, 1 mL of standardized bacterial suspension (5.0 McFarland) was added to each tube, to obtain a final bacterial concentration of ~ 10^8^ CFU/mL. Results were read by visual inspections after each 1 h of incubation (35-37 °C), for up to 4 h. Isolates were considered resistant to polymyxin B when color change (orange to yellow) was evidenced in both tubes (Fig. 1. A).

For the miniaturized version, RCPEm, 2 disks of polymyxin B 300 IU (Oxoid) were added in 15 mL of NP solution, kept at room temperature for 30 min, and at 35–37 °C for another 4 h, in order to complete the antibiotic elution from the disk to the broth, reproducing the full incubation period of PBDE [36]. Isolates were evaluated in microtiter plates, where 150 µL of antibiotic-free NP solution was pipetted in one well, and in another well, 150 µL of NP solution containing the previously eluted antibiotic. Then, 50 µL of a standardized bacterial suspension (3.0 McFarland) was inoculated into each well, reaching a final bacterial concentration of ~ 10^8^ CFU/mL. After adding bacterial suspension, the well-containing antibiotic had a final concentration of 3 µg/mL of polymyxin B. Plates were incubated and read visually each 1 h, for up to 4 h. Color change of both wells (growth control and test) from orange to yellow indicated resistance to polymyxin B (Fig. 1. B).

## Results

**Fig. 2:**
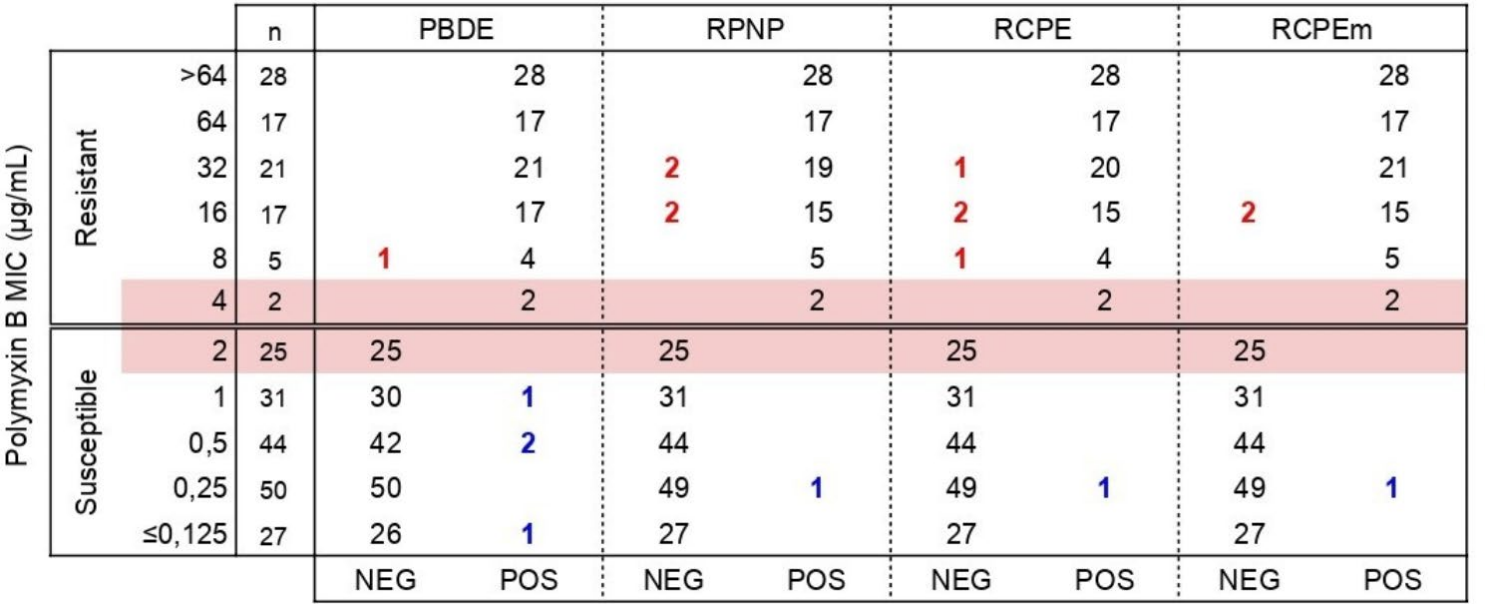
Results of PBDE, RPNP, RCPE and RCPEm for the detection of susceptibility to polymyxin B among *Enterobacterales* MIC, minimum inhibitory concentration; PBDE, polymyxin B broth disk elution; RPNP, rapid polymyxin NP test; RCPE, rapid colorimetric polymyxin B elution; RCPEm, rapid colorimetric polymyxin B microelution; R, Resistant; S, susceptible

MIC, minimum inhibitory concentration; n, number of isolates tested; PBDE, polymyxin B broth disk elution; RPNP, rapid polymyxin NP test; RCPE, rapid colorimetric polymyxin B elution; RCPEm, rapid colorimetric polymyxin B microelution; POS, positive; NEG, negative. Red numbers: false negative (very major error); Blue numbers: false positive (major error). Highlighted Lines (pink): Borderline MICs.

According to BMD, 33.7% (90/267) of *Enterobacterales* were resistant to polymyxin B, including 18 isolates intrinsically resistant (*S. marcescens*, *P. mirabilis*, *P. rettgeri* and *P. stuartii.* MICs ranged from ≤ 0.125 µg/mL to > 64 µg/mL, with 10.1% (27/267) of isolates presenting borderline MICs (2 or 4 µg/mL), as shown in Fig. 2.

Table 1 presents the performance of methodologies compared to BMD and Table 2 details discrepant results. All tests presented sensitivity, specificity, and major error (ME) within required values (i.e., > 95%, > 95%, and < 3%, respectively), according to FDA [39]. On the other hand, RPNP and RCPE had 4.4% of very major errors (VME). Of note, RCPE performed identically to RPNP with the same isolates being considered false positive and false negative by both methodologies. Results of the miniaturized version, RCPEm, were similar to RCPE, but categorical agreement (CA) and sensitivity (98.9% and 97.8%, respectively) were higher than RCPE, with VME (2.2%) less frequent (2 *K. pneumoniae* MIC 16 µg/mL).

**Table 1:** Performance of methodologies to detect polymyxin B resistance among *Enterobacterales* compared to broth microdilution

	CA	Sensitivity	Specificity	PPV	NPV	ME	VME
PBDE	98.1%	98.9%	97.7%	95.7%	99.4%	2.3%	1.1%
RPNP	98.1%	95.6%	99.4%	98.9%	97.8%	0.6%	4.4%
RCPE	98.1%	95.6%	99.4%	98.9%	97.8%	0.6%	4.4%
RCPEm	98.9%	97.8%	99.4%	98.9%	98.9%	0.6%	2.2%

PBDE, polymyxin B broth disk elution; RPNP, rapid polymyxin NP test; RCPE, rapid colorimetric polymyxin B elution; RCPEm, rapid colorimetric polymyxin B microelution; CA, categorical agreement; PPV, positive predictive value; NPV, negative predictive value; ME, major error; VME, very major error

**Table 2:** Discrepancies observed among *Enterobacterales* with PBDE, RPNP, RCPE, and RCPEm.

Species	MIC^a^ (µg/mL)	PBDE	RPNP	RCPE	RCPEm	Categorical error
*E. cloacae* complex	≤ 0.125	**R** ^**b**^	S	S	S	Major
*K. pneumoniae*	0,25	S	**R**	**R**	**R**	
*K. pneumoniae*	0,5	**R**	S	S	S	
*E. coli*	0,5	**R**	S	S	S	
*K. pneumoniae*	1	**R**	S	S	S	
*K. pneumoniae*	8	**S**	R	**S**	R	Very major
*K. pneumoniae*	16	R	**S**	**S**	**S**	
*K. pneumoniae*	16	R	**S**	**S**	**S**	
*E. cloacae* complex	32	R	**S**	R	R	
*E. cloacae* complex	32	R	**S**	**S**	R	

^a^ Minimum inhibitory concentration of Polymyxin B determined by Broth Microdilution

^b^ Discrepant results are highlighted in bold

MIC, minimum inhibitory concentration; PBDE, polymyxin B broth disk elution; RPNP, rapid polymyxin NP test; RCPE, rapid colorimetric polymyxin B elution; RCPEm, rapid colorimetric polymyxin B microelution; R, Resistant; S, susceptible

Noteworthy, RPNP identified 98.8% (85/86) of the truly positive (resistant) isolates within 2 h of incubation. Surprisingly, 3 h was requested for one *S. marcescens* (MIC > 64 µg/mL). On the other hand, only 13.3% of truly resistant isolates were positive after 2 h of incubation in RCPE. Indeed, most isolates (72.2%) demanded 3 h for color change. Overall, 91.0% (80/88) of true positive results in RCPEm were observed within 2 h, and only 4 isolates demanded 4 h for positive results (MIC 8 µg/mL [n = 1], 16 µg/mL [n = 2] and 32 µg/mL [ n = 1]) (supplementary table).

## Discussion

We observed good accuracy of the alternative methodologies (PBDE, RPNP, and, mainly, RCPE and RCPEm) in defining polymyxin B resistance in a diverse population of *Enterobacterale*s, which included a considerable percentage of isolates with borderline MICs.

CBDE/PBDE had proved to be simple, easy to perform, and cheap and was endorsed by CLSI [40]. Although in a limited number, studies presented satisfactory results [32, 33, 36, 41], with CA ranging from 91.2 to 99.5%, VME from 1.1 to 8% and ME from 0 to 12% when *Enterobacterales* were evaluated and our results are in accordance with these results.

Since its first publication, RPNP has been extensively evaluated in several locations, exhibiting sensitivity from 91.0 to 100% and specificity from 70.0 to 100% [22–31]. Our results corroborate this performance (95.6% sensitivity and 99.4% specificity). It is recognized that certain bacterial genera could negatively influence sensitivity and specificity of RPNP, as shown by Simar et al. (2017) [42] when evaluating exclusively *Enterobacter* spp., reaching only 25% of sensitivity. Belda-Orlowski et al. (2019) [26] also observed the influence of this species over test performance when they stratified bacterial population: the specificity of 70% for *Enterobacterales* overall was reduced to 30% when evaluating only *Enterobacter* spp. It is well recognized that heteroresistance to polymyxins, frequently expressed by *Enterobacter* spp., may justify, at least partially, these findings. Our population included 15.7% (42/267) of *E. cloacae* complex, mostly (95.2%; 40/42) susceptible to polymyxin B. Of note, both *E. cloacae* complex resistant to polymyxin B (MIC 32 µg/mL) presented false negative results in RPNP. Therefore, it seems very clear that our number of *Enterobacter* spp. resistant to polymyxins was not large enough to influence the performance of the test, which could be mentioned as a limitation of our study.

Considering RCPE, we introduced some modifications. The version proposed elsewhere [38] assessed polymyxins susceptibility by exposing them to a colistin concentration of 3.7 µg/ml, after elution from a 10 µg colistin disk into 2.7 ml of NP solution. The inoculum used by the authors was a 1µL loop (~ 10^8^ CFU/mL). On the other hand, we performed RCPE using 300 IU polymyxin B disks to obtain a concentration of 2 µg/ml (1 disk in 15 mL of NP solution), in order to maintain the same antibiotic concentration of the methodology approved by CLSI (CBDE). Besides, instead of using 1µL loop, we chose 1 mL of an adjusted bacterial suspension (5.0 McFarland) as bacterial inoculum (reaching the same final concentration), aiming to improve standardization.

Ngudsuntia et al. (2021) [38] found a sensitivity of 94.6% and 5.4% of VME, caused by 2 *K. pneumoniae* and 1 *E. cloacae* complex with false negative results, all with borderline MICs (4 µg/mL). Among our population, 4 isolates had false negative results, with a consequent sensitivity of 97.8% and VME of 4.4%. On the other hand, the authors of the aforementioned study found a false positive result (*K. pneumoniae*, MIC 2 µg/mL), reaching a specificity of 99.4% and ME of 0.6%, identical to our results. Furthermore, the miniaturized version we proposed (RCPEm) had better performance with greater sensitivity (97.8% vs. 95.6%), lower VME (2.2 vs. 4.4%), and higher CA (98.9 vs. 98.1%). The reduced volume needed for miniaturized version would demand less bacterial growth to color change, which could explain the superior performance.

Noteworthy, compared to BMD (18-24 h) and PBDE (16-20 h), RCPE and RCPEm were faster (up to 3 h and 2 h for most truly positive isolates, respectively), which is an advantage that must be highlighted. Negative results were defined after 4 h of incubation, enabling a fast and efficient therapy adjustment.

It should be noted that the percentage of VME observed by us is above the accepted value published by the FDA (1.11% for the number of isolates resistant we evaluated) [39]. However, the limited number of resistant isolates (n = 90) included in the study may explain, at least partially, the elevated percentage of VME observed.

One could expect that the RCPE and RCPEm would have better sensitivity than PBDE, as a color change would improve the reading of positive results. However, it was not observed by us, indicating that the major advantages of RCPE and RCPEm over PBDE are the higher specificity and the considerably reduced incubation time.

RCPE and RCPEm had different final concentrations of polymyxin B, 2 and 3 µg/mL, respectively. It could be a limitation when comparing the performance of both tests, especially in a population with a high percentage of borderline MICs. However, as the false-positive result we observed with both methodologies had MIC 0.25 µg/mL, we did not consider it as an issue. Interestingly, both methodologies had excellent performance considering those isolates with borderline MICs (100% CA among this population of 27 isolates).

The qualitative nature of RCPE and RCPEm could be considered a limitation of these methodologies. However, as the therapeutic window of polymyxins is very narrow, dose management is not a routine conduct in clinical practice. For this reason, results obtained by these methodologies, as well as PBDE, do not require, overall, confirmation by BMD [43].

One could mention that a disadvantage of RCPEm would be the need of preparing NP solution with the antibiotic eluted when performing the test. To simplify the methodology, we evaluated it using a solution previously prepared and stored (4–8 °C) for up to 30 days. The pre-eluted stored and the freshly prepared solution were evaluated at the same time, in the same plate with the same bacterial inoculum, with a subset of 7 clinical isolates. The results were fully concordant (supplementary table).

The predominance of *K. pneumoniae* over other species evaluated must be recognized as a limitation of our study. However, the different origins (4 different hospitals - supplementary table) of isolates and the prolonged period of recovery (2017–2022) reduces, at least partially, the probability of clonal relationship among these *K. pneumoniae*. Besides, although in less frequency, we included 10 other species trying to evaluate some eventual species-specific interference on the performance of methodologies. Moreover, it should be noted that this distribution of species represents the epidemiology of our region.

## Conclusion

The RCPE and RCPEm proved to be excellent alternatives for determining susceptibility to polymyxin B. Indeed, they demonstrated to be at least as accurate as those methodologies they are derived (RPNP and PBDE). Due to their lower cost, easier execution, and faster release of results compared to BMD, both methodologies could be routinely implemented in clinical laboratories. Storing the antibiotic-eluted solution may be an option, although this issue should be better evaluated. Because of the reduced volumes and lower incubation time for most isolates, RCPEm seems to adapt better to the routine of microbiology laboratories.

### Electronic supplementary material

Below is the link to the electronic supplementary material.

Supplementary Material 1

## Data Availability

All data generated or analysed during this study are included in this published article [and its supplementary information files].

## Abbreviations

CRE: Carbapenem-resistant
WHO: World Health Organization
CLSI: Clinical and Laboratory Standards Institute
EUCAST: European Committee on Antimicrobial Susceptibility Testing
FDA: Food and Drug Administration
BMD: Broth microdilution
RPNP: Rapid Polymyxin NP
CBDE: Colistin broth disk elution
PBDE: Polymyxin B broth disk elution
RCPE: Rapid colorimetric polymyxin B elution
RCPEm: Rapid colorimetric polymyxin B microelution
MIC: Minimum inhibitory concentration
ME: Major error
VME: Very major error
CA: Categorical agreement
PPV: Positive predictive value
NPV: Negative predictive value
