# Supplementary material for: Detection of polymyxins resistance among Enterobacterales: evaluation of available methods and proposal of a new rapid and feasible methodology
Source: Ann Clin Microbiol Antimicrob. 2023 Aug 10;22:71. doi: 10.1186/s12941-023-00618-7 (PMC10416366; doi:10.1186/s12941-023-00618-7)
Supplement: Supplementary file 1 — Supplementary Material 1 [file 12941_2023_618_MOESM1_ESM.docx]

Results of PBDE, RPNP, RCPE and RCPEm for the detection of susceptibility to polymyxin B among *Enterobacterales*.

| Isolate | Species | Hospital | Isolation  (MM/YYYY) | MIC^a^  (µg/mL) | PBDE | RPNP | | RCPE | | RCPEm | |
| --- | --- | --- | --- | --- | --- | --- | --- | --- | --- | --- | --- |
|  |  |  |  |  |  | Result | Positivity Time (h) | Result | Positivity Time (h) | Result | Positivity Time (h) |
| 145 | *K. pneumoniae* | A | 07/2018 | ≤0.125 | N | N | - | N | - | N | - |
| 170 | *K. pneumoniae* | A | 07/2018 | ≤0.125 | N | N | - | N | - | N | - |
| 179 | *K. pneumoniae* | A | 08/2018 | ≤0.125 | N | N | - | N | - | N | - |
| 181 | *E. cloacae complex* | A | 08/2018 | ≤0.125 | N | N | - | N | - | N | - |
| 187 | *K. oxytoca* | A | 08/2018 | ≤0.125 | N | N | - | N | - | N | - |
| 188 | *K. oxytoca* | A | 08/2018 | ≤0.125 | N | N | - | N | - | N | - |
| 189 | *K. pneumoniae* | A | 08/2018 | ≤0.125 | N | N | - | N | - | N | - |
| 193 | *E. coli* | A | 09/2018 | ≤0.125 | N | N | - | N | - | N | - |
| 201 | *K. pneumoniae* | A | 09/2018 | ≤0.125 | N | N | - | N | - | N** | - |
| 251 | *K. pneumoniae* | B | 06/2018 | ≤0.125 | N | N | - | N | - | N | - |
| 347 | *E. coli* | A | 04/2019 | ≤0.125 | N | N | - | N | - | N | - |
| 421 | *E. coli* | A | 07/2019 | ≤0.125 | N | N | - | N | - | N | - |
| 575 | *E. cloacae complex* | C | 06/2019 | ≤0.125 | N | N | - | N | - | N | - |
| 745 | *K. ozaenae* | A | 02/2020 | ≤0.125 | N | N | - | N | - | N | - |
| 751 | *E. coli* | A | 03/2020 | ≤0.125 | N | N | - | N | - | N | - |
| 766 | *E. coli* | A | 04/2020 | ≤0.125 | N | N | - | N | - | N | - |
| 774 | *E. cloacae complex* | A | 04/2020 | ≤0.125 | N | N | - | N | - | N | - |
| 779 | *C. freundii* | A | 05/2020 | ≤0.125 | N | N | - | N | - | N | - |
| 804 | *E. cloacae complex* | A | 06/2020 | ≤0.125 | N | N | - | N | - | N | - |
| 813 | *E. coli* | A | 06/2020 | ≤0.125 | N | N | - | N | - | N | - |
| 828 | *E. cloacae complex* | A | 07/2020 | ≤0.125 | N | N | - | N | - | N | - |
| 902 | *E. cloacae complex* | C | 08/2020 | ≤0.125 | P | N | - | N | - | N | - |
| 936 | *E. cloacae complex* | C | 09/2020 | ≤0.125 | N | N | - | N | - | N | - |
| 946 | *E. cloacae complex* | C | 09/2020 | ≤0.125 | N | N | - | N | - | N | - |
| 955 | *E. cloacae complex* | C | 10/2020 | ≤0.125 | N | N | - | N | - | N | - |
| 1262 | *K. pneumoniae* | D | 04/2022 | ≤0.125 | N | N | - | N | - | N | - |
| 1270 | *K. pneumoniae* | D | 04/2022 | ≤0.125 | N | N | - | N | - | N | - |
| 146 | *K. pneumoniae* | A | 07/2018 | 0.25 | N | N | - | N | - | N | - |
| 147 | *K. pneumoniae* | A | 07/2018 | 0.25 | N | N | - | N | - | N | - |
| 151 | *K. pneumoniae* | A | 07/2018 | 0.25 | N | N | - | N | - | N | - |
| 165 | *K. pneumoniae* | A | 07/2018 | 0.25 | N | N | - | N | - | N | - |
| 185 | *K. pneumoniae* | A | 08/2018 | 0.25 | N | N | - | N | - | N | - |
| 191 | *K. pneumoniae* | A | 09/2018 | 0.25 | N | N | - | N | - | N | - |
| 196 | *K. pneumoniae* | A | 09/2018 | 0.25 | N | N | - | N | - | N | - |
| 203 | *E. cloacae complex* | A | 09/2018 | 0.25 | N | N | - | N | - | N** | - |
| 205 | *K. pneumoniae* | A | 09/2018 | 0.25 | N | N | - | N | - | N | - |
| 246 | *C. freundii* | B | 07/2018 | 0.25 | N | N | - | N | - | N | - |
| 267 | *K. pneumoniae* | B | 08/2017 | 0.25 | N | P | 3 | I | - | P | 3 |
| 270 | *K. pneumoniae* | B | 08/2017 | 0.25 | N | N | - | N | - | N | - |
| 289 | *K. oxytoca* | B | 08/2018 | 0.25 | N | N | - | N | - | N | - |
| 302 | *K. pneumoniae* | C | 05/2019 | 0.25 | N | N | - | N | - | N | - |
| 369 | *E. cloacae complex* | A | 06/2019 | 0.25 | N | N | - | N | - | N | - |
| 563 | *E. cloacae complex* | C | 08/2018 | 0.25 | N | N | - | N | - | N | - |
| 570 | *E. coli* | C | 05/2019 | 0.25 | N | N | - | N | - | N | - |
| 576 | *K. oxytoca* | C | 05/2019 | 0.25 | N | N | - | N | - | N | - |
| 597 | *E. cloacae complex* | A | 09/2019 | 0.25 | N | N | - | N | - | N | - |
| 642 | *K. aerogenes* | A | 11/2019 | 0.25 | N | N | - | N | - | N | - |
| 716 | *K. oxytoca* | A | 01/2020 | 0.25 | N | N | - | N | - | N | - |
| 746 | *E. coli* | A | 02/2020 | 0.25 | N | N | - | N | - | N | - |
| 771 | *C. freundii* | A | 04/2020 | 0.25 | N | N | - | N | - | N | - |
| 777 | *E. coli* | A | 04/2020 | 0.25 | N | N | - | N | - | N | - |
| 794 | *C. freundii* | A | 05/2020 | 0.25 | N | N | - | N | - | N | - |
| 795 | *E. coli* | A | 05/2020 | 0.25 | N | N | - | N | - | N | - |
| 838 | *K. ozaenae* | A | 07/2020 | 0.25 | N | N | - | N | - | N | - |
| 911 | *K. aerogenes* | C | 10/2020 | 0.25 | N | N | - | N | - | N | - |
| 923 | *C. freundii* | C | 08/2020 | 0.25 | N | N | - | N | - | N | - |
| 925 | *E. cloacae complex* | C | 08/2020 | 0.25 | N | N | - | N | - | N | - |
| 939 | *E. cloacae complex* | C | 08/2020 | 0.25 | N | N | - | N | - | N | - |
| 970 | *E. coli* | A | 08/2020 | 0.25 | N | N | - | N | - | N | - |
| 1005 | *E. cloacae complex* | A | 10/2020 | 0.25 | N | N | - | N | - | N | - |
| 1083 | *E. cloacae complex* | C | 06/2021 | 0.25 | N | N | - | N | - | N | - |
| 1224 | *K. pneumoniae* | D | 03/2022 | 0.25 | N | N | - | N | - | N | - |
| 1226 | *K. pneumoniae* | D | 03/2022 | 0.25 | N | N | - | N | - | N | - |
| 1228 | *K. pneumoniae* | D | 03/2022 | 0.25 | N | N | - | N | - | N | - |
| 1230 | *E. cloacae complex* | D | 03/2022 | 0.25 | N | N | - | N | - | N | - |
| 1231 | *K. pneumoniae* | D | 03/2022 | 0.25 | N | N | - | N | - | N | - |
| 1235 | *E. coli* | D | 03/2022 | 0.25 | N | N | - | N | - | N | - |
| 1241 | *K. pneumoniae* | D | 03/2022 | 0.25 | N | N | - | N | - | N | - |
| 1248 | *K. pneumoniae* | D | 03/2022 | 0.25 | N | N | - | N | - | N | - |
| 1249 | *K. pneumoniae* | D | 03/2022 | 0.25 | N | N | - | N | - | N | - |
| 1253 | *K. pneumoniae* | D | 03/2022 | 0.25 | N | N | - | N | - | N | - |
| 1256 | *K. pneumoniae* | D | 03/2022 | 0.25 | N | N | - | N | - | N | - |
| 1261 | *K. pneumoniae* | D | 03/2022 | 0.25 | N | N | - | N | - | N | - |
| 1266 | *K. pneumoniae* | D | 04/2022 | 0.25 | N | N | - | N | - | N | - |
| 1280 | *K. pneumoniae* | D | 07/2022 | 0.25 | N | N | - | N | - | N | - |
| 1285 | *K. pneumoniae* | D | 07/2022 | 0.25 | N | N | - | N | - | N | - |
| 1290 | *K. pneumoniae* | D | 07/2022 | 0.25 | N | N | - | N | - | N | - |
| 150 | *K. pneumoniae* | A | 07/2018 | 0.5 | N | N | - | N | - | N | - |
| 175 | *K. pneumoniae* | A | 08/2018 | 0.5 | N | N | - | N | - | N | - |
| 176 | *E. cloacae complex* | A | 08/2018 | 0.5 | N | N | - | N | - | N | - |
| 178 | *E. coli* | A | 08/2018 | 0.5 | N | N | - | N | - | N | - |
| 198 | *K. pneumoniae* | A | 09/2018 | 0.5 | N | N | - | N | - | N | - |
| 204 | *E. coli* | A | 09/2018 | 0.5 | N | N | - | N | - | N** | - |
| 211 | *K. pneumoniae* | A | 12/2018 | 0.5 | N | N | - | N | - | N | - |
| 277 | *K. pneumoniae* | B | 09/2018 | 0.5 | N | N | - | N | - | N | - |
| 300 | *K. pneumoniae* | C | 11/2018 | 0.5 | N | N | - | N | - | N | - |
| 325 | *E. cloacae complex* | C | 02/2019 | 0.5 | N | N | - | N | - | N | - |
| 336 | *E. cloacae complex* | C | 12/2019 | 0.5 | N | N | - | N | - | N | - |
| 359 | *E. coli* | A | 05/2019 | 0.5 | N | N | - | N | - | N | - |
| 392 | *K. pneumoniae* | A | 06/2019 | 0.5 | N | N | - | N | - | N | - |
| 393 | *K. pneumoniae* | A | 06/2019 | 0.5 | N | N | - | N | - | N | - |
| 403 | *K. pneumoniae* | A | 07/2019 | 0.5 | N | N | - | N | - | N | - |
| 406 | *K. pneumoniae* | A | 07/2019 | 0.5 | N | N | - | N | - | N | - |
| 615 | *E. coli* | A | 09/2019 | 0.5 | N | N | - | N | - | N | - |
| 682 | *E. coli* | A | 12/2019 | 0.5 | N | N | - | N | - | N | - |
| 807 | *E. coli* | A | 06/2020 | 0.5 | N | N | - | N | - | N | - |
| 900 | *K. aerogenes* | C | 06/2020 | 0.5 | N | N | - | N | - | N | - |
| 921 | *E. cloacae complex* | C | 08/2020 | 0.5 | N | N | - | N | - | N | - |
| 926 | *E. cloacae complex* | C | 08/2020 | 0.5 | N | N | - | N | - | N | - |
| 941 | *E. cloacae complex* | C | 08/2020 | 0.5 | N | N | - | N | - | N | - |
| 944 | *E. cloacae complex* | C | 08/2020 | 0.5 | N | N | - | N | - | N | - |
| 963 | *E. cloacae complex* | C | 11/2020 | 0.5 | N | N | - | N | - | N | - |
| 985 | *K. aerogenes* | A | 09/2020 | 0.5 | N | N | - | N | - | N | - |
| 1068 | *E. coli* | A | 02/2021 | 0.5 | N | N | - | N | - | N | - |
| 1223 | *K. pneumoniae* | D | 03/2022 | 0.5 | N | N | - | N | - | N | - |
| 1238 | *E. coli* | D | 03/2022 | 0.5 | N | N | - | N | - | N | - |
| 1242 | *K. pneumoniae* | D | 03/2022 | 0.5 | N | N | - | N | - | N | - |
| 1243 | *E. coli* | D | 03/2022 | 0.5 | N | N | - | N | - | N | - |
| 1244 | *K. pneumoniae* | D | 03/2022 | 0.5 | N | N | - | N | - | N | - |
| 1246 | *K. pneumoniae* | D | 03/2022 | 0.5 | N | N | - | N | - | N | - |
| 1247 | *K. pneumoniae* | D | 03/2022 | 0.5 | P | N | - | N | - | N | - |
| 1250 | *E. coli* | D | 04/2022 | 0.5 | P | N | - | N | - | N | - |
| 1251 | *K. pneumoniae* | D | 04/2022 | 0.5 | N | N | - | N | - | N | - |
| 1252 | *K. pneumoniae* | D | 04/2022 | 0.5 | N | N | - | N | - | N | - |
| 1258 | *K. pneumoniae* | D | 03/2022 | 0.5 | N | N | - | N | - | N | - |
| 1259 | *K. pneumoniae* | D | 03/2022 | 0.5 | N | N | - | N | - | N | - |
| 1263 | *K. pneumoniae* | D | 04/2022 | 0.5 | N | N | - | N | - | N | - |
| 1265 | *E. coli* | D | 04/2022 | 0.5 | N | N | - | N | - | N | - |
| 1274 | *K. pneumoniae* | D | 03/2022 | 0.5 | N | N | - | N | - | N | - |
| 1275 | *K. pneumoniae* | D | 07/2022 | 0.5 | N | N | - | N | - | N | - |
| 1277 | *K. pneumoniae* | D | 07/2022 | 0.5 | N | N | - | N | - | N | - |
| 160 | *K. pneumoniae* | A | 07/2018 | 1 | N | N | - | N | - | N | - |
| 294 | *K. oxytoca* | C | 12/2018 | 1 | N | N | - | N | - | N | - |
| 295 | *K. pneumoniae* | C | 12/2018 | 1 | N | N | - | N | - | N | - |
| 298 | *K. pneumoniae* | C | 11/2018 | 1 | N | N | - | N | - | N | - |
| 301 | *K. pneumoniae* | C | 11/2018 | 1 | N | N | - | N | - | N | - |
| 323 | *E. cloacae complex* | C | 02/2019 | 1 | N | N | - | N | - | N | - |
| 332 | *E. coli* | C | 01/2019 | 1 | N | N | - | N | - | N | - |
| 342 | *C. freundii* | C | 11/2018 | 1 | N | N | - | N | - | N | - |
| 366 | *K. pneumoniae* | A | 05/2019 | 1 | N | N | - | N | - | N | - |
| 376 | *E. cloacae complex* | A | 06/2019 | 1 | N | N | - | N | - | N | - |
| 384 | *K. pneumoniae* | A | 06/2019 | 1 | N | N | - | N | - | N | - |
| 396 | *K. pneumoniae* | A | 06/2019 | 1 | N | N | - | N | - | N | - |
| 437 | *K. pneumoniae* | A | 07/2019 | 1 | N | N | - | N | - | N | - |
| 448 | *K. pneumoniae* | A | 07/2019 | 1 | N | N | - | N | - | N | - |
| 553 | *K. oxytoca* | C | 03/2019 | 1 | N | N | - | N | - | N | - |
| 589 | *E. cloacae complex* | A | 08/2019 | 1 | N | N | - | N | - | N | - |
| 770 | *K. ozaenae* | A | 04/2020 | 1 | N | N | - | N | - | N | - |
| 772 | *K. oxytoca* | A | 04/2020 | 1 | N | N | - | N | - | N | - |
| 910 | *E. cloacae complex* | C | 10/2020 | 1 | N | N | - | N | - | N | - |
| 948 | *E. coli* | C | 08/2020 | 1 | N | N | - | N | - | N | - |
| 949 | *E. cloacae complex* | C | 10/2020 | 1 | N | N | - | N | - | N | - |
| 950 | *E. cloacae complex* | C | 09/2020 | 1 | N | N | - | N | - | N | - |
| 954 | *E. cloacae complex* | C | 10/2020 | 1 | N | N | - | N | - | N | - |
| 1061 | *E. cloacae complex* | A | 01/2021 | 1 | N | N | - | N | - | N | - |
| 1234 | *K. pneumoniae* | D | 03/2022 | 1 | P | N | - | N | - | N | - |
| 1236 | *K. pneumoniae* | D | 03/2022 | 1 | N | N | - | N | - | N | - |
| 1264 | *K. pneumoniae* | D | 04/2022 | 1 | N | N | - | N | - | N | - |
| 1269 | *K. pneumoniae* | D | 04/2022 | 1 | N | N | - | N | - | N | - |
| 1273 | *K. pneumoniae* | D | 03/2022 | 1 | N | N | - | N | - | N | - |
| 1284 | *K. pneumoniae* | D | 07/2022 | 1 | N | N | - | N | - | N | - |
| 1289 | *K. pneumoniae* | D | 07/2022 | 1 | N | N | - | N | - | N | - |
| 186 | *K. pneumoniae* | A | 08/2018 | 2* | N | N | - | N | - | N | - |
| 194 | *K. pneumoniae* | A | 09/2018 | 2* | N | N | - | N | - | N | - |
| 256 | *K. pneumoniae* | B | 07/2018 | 2* | N | N | - | N | - | N | - |
| 264 | *K. pneumoniae* | B | 08/2017 | 2* | N | N | - | N | - | N | - |
| 284 | *K. oxytoca* | B | 05/2018 | 2* | N | N | - | N | - | N | - |
| 291 | *K. pneumoniae* | B | 12/2017 | 2* | N | N | - | N | - | N | - |
| 296 | *K. pneumoniae* | C | 12/2018 | 2* | N | N | - | N | - | N | - |
| 303 | *K. pneumoniae* | C | 05/2019 | 2* | N | N | - | N | - | N | - |
| 305 | *E. cloacae complex* | C | 04/2019 | 2* | N | N | - | N | - | N | - |
| 307 | *C. freundii* | C | 04/2019 | 2* | N | N | - | N | - | N | - |
| 314 | *E. cloacae complex* | C | 04/2019 | 2* | N | N | - | N | - | N | - |
| 317 | *E. cloacae complex* | C | 03/2019 | 2* | N | N | - | N | - | N | - |
| 322 | *C. freundii* | C | 02/2019 | 2* | N | N | - | N | - | N | - |
| 375 | *K. pneumoniae* | A | 06/2019 | 2* | N | N | - | N | - | N | - |
| 467 | *K. pneumoniae* | A | 08/2019 | 2* | N | N | - | N | - | N** | - |
| 522 | *K. pneumoniae* | C | 08/2015 | 2* | N | N | - | N | - | N | - |
| 523 | *K. pneumoniae* | C | 10/2015 | 2* | N | N | - | N | - | N | - |
| 524 | *K. pneumoniae* | C | 06/2019 | 2* | N | N | - | N | - | N | - |
| 543 | *K. pneumoniae* | C | 06/2019 | 2* | N | N | - | N | - | N | - |
| 554 | *K. pneumoniae* | C | 07/2019 | 2* | N | N | - | N | - | N | - |
| 690 | *E. cloacae complex* | A | 12/2019 | 2* | N | N | - | N | - | N | - |
| 747 | *E. cloacae complex* | A | 03/2020 | 2* | N | N | - | N | - | N | - |
| 1033 | *E. cloacae complex* | A | 12/2020 | 2* | N | N | - | N | - | N | - |
| 1229 | *K. pneumoniae* | D | 03/2022 | 2* | N | N | - | N | - | N | - |
| 1272 | *K. pneumoniae* | D | 04/2022 | 2* | N | N | - | N | - | N | - |
| 199 | *K. pneumoniae* | A | 09/2018 | 4* | P | P | 2 | P | 4 | P** | 2 |
| 292 | *K. pneumoniae* | B | 02/2018 | 4* | P | P | 2 | P | 3 | P | 1 |
| 212 | *K. pneumoniae* | A | 11/2018 | 8 | P | P | 2 | P | 2 | P | 4 |
| 258 | *K. pneumoniae* | B | 07/2018 | 8 | P | P | 2 | P | 2 | P | 2 |
| 266 | *K. pneumoniae* | B | 08/2017 | 8 | P | P | 2 | P | 3 | P | 2 |
| 293 | *K. pneumoniae* | B | 02/2018 | 8 | P | P | 2 | P | 3 | P | 2 |
| 1276 | *K. pneumoniae* | D | 07/2022 | 8 | N | P | 2 | N | - | P | 2 |
| 144 | *K. pneumoniae* | A | 07/2018 | 16 | P | P | 1 | P | 3 | P | 2 |
| 169 | *K. pneumoniae* | A | 07/2018 | 16 | P | P | 2 | P | 3 | P | 2 |
| 285 | *K. pneumoniae* | B | 08/2018 | 16 | P | P | 2 | P | 4 | P | 4 |
| 287 | *K. pneumoniae* | B | 08/2018 | 16 | P | P | 2 | P | 4 | P | 4 |
| 288 | *K. pneumoniae* | B | 08/2018 | 16 | P | P | 2 | P | 3 | P | 3 |
| 320 | *K. pneumoniae* | C | 03/2019 | 16 | P | P | 2 | P | 3 | P | 2 |
| 382 | *K. pneumoniae* | A | 07/2019 | 16 | P | P | 2 | P | 3 | P | 2 |
| 418 | *K. pneumoniae* | A | 07/2019 | 16 | P | P | 2 | P | 3 | P | 2 |
| 420 | *K. pneumoniae* | A | 04/2019 | 16 | P | P | 2 | P | 3 | P | 2 |
| 422 | *K. pneumoniae* | A | 07/2019 | 16 | P | P | 2 | P | 2 | P | 2 |
| 442 | *K. pneumoniae* | A | 08/2019 | 16 | P | P | 2 | P | 3 | P | 2 |
| 473 | *K. pneumoniae* | A | 08/2019 | 16 | P | P | 2 | P | 3 | P | 2 |
| 835 | *P. mirabilis* | A | 07/2020 | 16 | P | P | 2 | P | 2 | P | 2 |
| 1255 | *K. pneumoniae* | D | 03/2022 | 16 | P | N | - | N | - | N | - |
| 1257 | *K. pneumoniae* | D | 03/2022 | 16 | P | N | - | N | - | N | - |
| 1271 | *K. pneumoniae* | D | 04/2022 | 16 | P | P | 2 | P | 3 | P | 2 |
| 1278 | *K. pneumoniae* | D | 07/2022 | 16 | P | P | 2 | P | 4 | P | 2 |
| 163 | *K. pneumoniae* | A | 07/2018 | 32 | P | P | 2 | P | 3 | P | 2 |
| 248 | *K. pneumoniae* | B | 07/2018 | 32 | P | P | 2 | P | 3 | P | 1 |
| 268 | *K. pneumoniae* | B | 08/2017 | 32 | P | P | 2 | P | 3 | P | 2 |
| 269 | *K. pneumoniae* | B | 08/2017 | 32 | P | P | 2 | P | 3 | P | 1 |
| 371 | *K. pneumoniae* | A | 06/2019 | 32 | P | P | 2 | P | 3 | P | 2 |
| 379 | *K. pneumoniae* | A | 06/2019 | 32 | P | P | 2 | P | 2 | P | 2 |
| 399 | *K. pneumoniae* | A | 06/2019 | 32 | P | P | 2 | P | 3 | P | 2 |
| 424 | *K. pneumoniae* | A | 07/2019 | 32 | P | P | 2 | P | 3 | P | 2 |
| 427 | *K. pneumoniae* | A | 07/2019 | 32 | P | P | 2 | P | 3 | P | 2 |
| 428 | *K. pneumoniae* | A | 07/2019 | 32 | P | P | 2 | P | 3 | P | 2 |
| 441 | *K. pneumoniae* | A | 07/2019 | 32 | P | P | 2 | P | 3 | P | 2 |
| 446 | *K. pneumoniae* | A | 08/2019 | 32 | P | P | 2 | P | 3 | P** | 1 |
| 450 | *K. pneumoniae* | A | 08/2019 | 32 | P | P | 2 | P | 3 | P | 2 |
| 454 | *K. pneumoniae* | A | 08/2019 | 32 | P | P | 2 | P | 3 | P | 2 |
| 457 | *K. pneumoniae* | A | 08/2019 | 32 | P | P | 2 | P | 3 | P | 2 |
| 468 | *K. pneumoniae* | A | 08/2019 | 32 | P | P | 2 | P | 3 | P | 2 |
| 474 | *K. pneumoniae* | A | 08/2019 | 32 | P | P | 2 | P | 3 | P | 2 |
| 908 | *E. cloacae complex* | C | 10/2020 | 32 | P | N | - | I | - | P | 2 |
| 913 | *E. cloacae complex* | C | 11/2020 | 32 | P | N | - | N | - | P | 2 |
| 1227 | *K. pneumoniae* | D | 03/2022 | 32 | P | P | 2 | P | 2 | P | 2 |
| 1254 | *E. coli* | D | 03/2022 | 32 | P | P | 2 | P | 3 | P | 4 |
| 407 | *K. pneumoniae* | A | 07/2019 | 64 | P | P | 2 | P | 2 | P | 1 |
| 412 | *K. pneumoniae* | A | 07/2019 | 64 | P | P | 2 | P | 3 | P | 2 |
| 416 | *K. pneumoniae* | A | 07/2019 | 64 | P | P | 2 | P | 3 | P | 2 |
| 423 | *K. pneumoniae* | A | 07/2019 | 64 | P | P | 2 | P | 4 | P | 2 |
| 425 | *K. pneumoniae* | A | 07/2019 | 64 | P | P | 2 | P | 3 | P | 2 |
| 429 | *K. pneumoniae* | A | 07/2019 | 64 | P | P | 2 | P | 3 | P | 2 |
| 439 | *K. pneumoniae* | A | 07/2019 | 64 | P | P | 2 | P | 3 | P | 2 |
| 458 | *K. pneumoniae* | A | 08/2019 | 64 | P | P | 2 | P | 3 | P | 2 |
| 461 | *K. pneumoniae* | A | 08/2019 | 64 | P | P | 2 | P | 3 | P | 1 |
| 1233 | *K. pneumoniae* | D | 03/2022 | 64 | P | P | 2 | P | 2 | P | 2 |
| 1239 | *K. pneumoniae* | D | 03/2022 | 64 | P | P | 2 | P | 3 | P | 2 |
| 1240 | *K. pneumoniae* | D | 03/2022 | 64 | P | P | 2 | P | 3 | P | 2 |
| 1281 | *K. pneumoniae* | D | 07/2022 | 64 | P | P | 2 | P | 3 | P | 2 |
| 1282 | *K. pneumoniae* | D | 07/2022 | 64 | P | P | 2 | P | 3 | P | 2 |
| 1283 | *K. pneumoniae* | D | 07/2022 | 64 | P | P | 2 | P | 3 | P | 2 |
| 1286 | *K. pneumoniae* | D | 07/2022 | 64 | P | P | 2 | P | 3 | P | 2 |
| 1287 | *K. pneumoniae* | D | 07/2022 | 64 | P | P | 2 | P | 3 | P | 2 |
| 154 | *K. pneumoniae* | A | 07/2018 | >64 | P | P | 2 | P | 3 | P | 2 |
| 180 | *S. marcescens* | A | 08/2018 | >64 | P | P | 3 | P | 4 | P | 2 |
| 377 | *K. pneumoniae* | A | 06/2019 | >64 | P | P | 2 | P | 2 | P | 2 |
| 389 | *K. pneumoniae* | A | 06/2019 | >64 | P | P | 2 | P | 3 | P | 1 |
| 398 | *K. pneumoniae* | A | 06/2019 | >64 | P | P | 2 | P | 3 | P** | 1 |
| 515 | *K. pneumoniae* | C | 06/2016 | >64 | P | P | 2 | P | 2 | P | 2 |
| 558 | *E. coli* | C | 09/2019 | >64 | P | P | 2 | P | 2 | P | 2 |
| 671 | *P. mirabilis* | A | 12/2019 | >64 | P | P | 2 | P | 3 | P | 2 |
| 802 | *S. marcescens* | A | 06/2020 | >64 | P | P | 2 | P | 4 | P | 2 |
| 803 | *P. mirabilis* | A | 06/2020 | >64 | P | P | 2 | P | 3 | P | 3 |
| 816 | *P. mirabilis* | A | 06/2020 | >64 | P | P | 2 | P | 3 | P | 3 |
| 991 | *P. rettgeri* | A | 10/2020 | >64 | P | P | 2 | P | 3 | P | 2 |
| 1008 | *P. stuartii* | A | 10/2020 | >64 | P | P | 2 | P | 3 | P | 2 |
| 1013 | *P. rettgeri* | A | 11/2020 | >64 | P | P | 2 | P | 3 | P | 2 |
| 1022 | *S. marcescens* | A | 12/2020 | >64 | P | P | 2 | P | 3 | P | 2 |
| 1031 | *S. marcescens* | A | 12/2020 | >64 | P | P | 2 | P | 3 | P | 2 |
| 1067 | *S. marcescens* | A | 01/2021 | >64 | P | P | 2 | P | 4 | P | 2 |
| 1080 | *E. coli* | C | 03/2021 | >64 | P | P | 2 | P | 3 | P | 2 |
| 1081 | *S. marcescens* | C | 04/2021 | >64 | P | P | 2 | P | 3 | P | 2 |
| 1082 | *S. marcescens* | C | 04/2021 | >64 | P | P | 2 | P | 3 | P | 3 |
| 1085 | *S. marcescens* | C | 05/2021 | >64 | P | P | 2 | P | 3 | P | 2 |
| 1095 | *S. marcescens* | C | 06/2021 | >64 | P | P | 2 | P | 3 | P | 2 |
| 1225 | *K. pneumoniae* | D | 03/2022 | >64 | P | P | 2 | P | 2 | P | 2 |
| 1232 | *K. pneumoniae* | D | 03/2022 | >64 | P | P | 2 | P | 2 | P | 2 |
| 1237 | *S. marcescens* | D | 03/2022 | >64 | P | P | 2 | P | 3 | P | 2 |
| 1260 | *K. pneumoniae* | D | 04/2022 | >64 | P | P | 2 | P | 3 | P | 2 |
| 1267 | *K. pneumoniae* | D | 04/2022 | >64 | P | P | 2 | P | 3 | P | 2 |
| 1279 | *K. pneumoniae* | D | 07/2022 | >64 | P | P | 2 | P | 3 | P | 2 |

^a^ Minimum inhibitory concentration of Polymyxin B determined by BMD

* MIC’s borderline

** Results also obtained using a previously prepared solution and stored (4-8°C) for up to 30 days.

MIC, minimum inhibitory concentration; PBDE, polymyxin B broth disk elution; RPNP, rapid polymyxin NP test; RCPE, rapid colorimetric polymyxin B elution; RCPEm, rapid colorimetric polymyxin B microelution; P, positive growth (resistant); N, negative growth (susceptible); I, indefinite growth.

A: Hospital A (generalist private hospital);

B: Hospital B (philanthropic hospital complex);

C: Hospital C (general public university hospital);

D: Hospital D (public hospital group).
